# Lung function and microbiota diversity in cystic fibrosis

**DOI:** 10.1186/s40168-020-00810-3

**Published:** 2020-04-02

**Authors:** Leah Cuthbertson, Alan W. Walker, Anna E. Oliver, Geraint B. Rogers, Damian W. Rivett, Thomas H. Hampton, Alix Ashare, J. Stuart Elborn, Anthony De Soyza, Mary P. Carroll, Lucas R. Hoffman, Clare Lanyon, Samuel M. Moskowitz, George A. O’Toole, Julian Parkhill, Paul J. Planet, Charlotte C. Teneback, Michael M. Tunney, Jonathan B. Zuckerman, Kenneth D. Bruce, Christopher J. van der Gast

**Affiliations:** 1 National Heart and Lung Institute, Imperial College London, London, UK; 2 Rowett Institute, University of Aberdeen, Aberdeen, UK; 3 Wellcome Sanger Institute, Hinxton, Cambridge, UK; 4 NERC Centre for Ecology & Hydrology, Wallingford, UK; 5 South Australian Health and Medical Research Institute, Adelaide, Australia; 6 School of Medicine, Flinders University, Adelaide, Australia; 7 Department of Natural Sciences, Manchester Metropolitan University, Manchester, UK; 8 Department of Microbiology and Immunology, Geisel School of Medicine at Dartmouth, Hanover, NH USA; 9 Department of Medicine, Dartmouth Hitchcock Medical Center, Lebanon, NH USA; 10 Adult Cystic Fibrosis Department, Royal Brompton Hospital, London, UK; 11 School of Medicine, Dentistry and Biomedical Sciences, Institute for Health Sciences, Queen’s University Belfast, Belfast, UK; 12 Institute of Cellular Medicine, NIHR Biomedical Research Centre for Ageing, Newcastle University, Newcastle, UK; 13 Department of Respiratory Medicine, Freeman Hospital, Newcastle, UK; 14 Cystic Fibrosis Unit, Southampton University Hospitals NHS Trust, Southampton, UK; 15 Seattle Children’s Hospital, Seattle, WA USA; 16 Departments of Pediatrics and Microbiology, University of Washington, Seattle, WA USA; 17 Faculty of Health and Life Sciences, University of Northumbria, Newcastle, UK; 18 Department of Pediatrics, Massachusetts General Hospital and Harvard Medical School, Boston, USA; 19 Vertex Pharmaceuticals, Boston, MA USA; 20 Department of Veterinary Medicine, University of Cambridge, Cambridge, UK; 21 Pediatric Infectious Disease Division, Children’s Hospital of Philadelphia, Philadelphia, PA USA; 22 Department of Pediatrics, Perelman School of Medicine, University of Pennsylvania, Philadelphia, PA USA; 23 Sackler Institute of Comparative Genomics, American Museum of Natural History, New York, NY USA; 24 Larner College of Medicine, University of Vermont, Burlington, VT USA; 25 School of Pharmacy, Queen’s University Belfast, Belfast, UK; 26 Maine Medical Center, Portland, ME USA; 27 School of Medicine, Tufts University, Boston, MA USA; 28 Institute of Pharmaceutical Science, King’s College London, London, UK; 29 Department of Life Sciences, Manchester Metropolitan University, Manchester, UK

**Keywords:** Cystic fibrosis, Lung function, Lung microbiota, Lung microbiome, Disease severity, Ecological patterns, Microbial surveillance, Biogeography, Antibiotics

## Abstract

**Background:**

Chronic infection and concomitant airway inflammation is the leading cause of morbidity and mortality for people living with cystic fibrosis (CF). Although chronic infection in CF is undeniably polymicrobial, involving a lung microbiota, infection surveillance and control approaches remain underpinned by classical aerobic culture-based microbiology. How to use microbiomics to direct clinical management of CF airway infections remains a crucial challenge. A pivotal step towards leveraging microbiome approaches in CF clinical care is to understand the ecology of the CF lung microbiome and identify ecological patterns of CF microbiota across a wide spectrum of lung disease. Assessing sputum samples from 299 patients attending 13 CF centres in Europe and the USA, we determined whether the emerging relationship of decreasing microbiota diversity with worsening lung function could be considered a generalised pattern of CF lung microbiota and explored its potential as an informative indicator of lung disease state in CF.

**Results:**

We tested and found decreasing microbiota diversity with a reduction in lung function to be a significant ecological pattern. Moreover, the loss of diversity was accompanied by an increase in microbiota dominance. Subsequently, we stratified patients into lung disease categories of increasing disease severity to further investigate relationships between microbiota characteristics and lung function, and the factors contributing to microbiota variance. Core taxa group composition became highly conserved within the severe disease category, while the rarer satellite taxa underpinned the high variability observed in the microbiota diversity. Further, the lung microbiota of individual patient were increasingly dominated by recognised CF pathogens as lung function decreased. Conversely, other bacteria, especially obligate anaerobes, increasingly dominated in those with better lung function. Ordination analyses revealed lung function and antibiotics to be main explanators of compositional variance in the microbiota and the core and satellite taxa. Biogeography was found to influence acquisition of the rarer satellite taxa.

**Conclusions:**

Our findings demonstrate that microbiota diversity and dominance, as well as the identity of the dominant bacterial species, in combination with measures of lung function, can be used as informative indicators of disease state in CF.

**Video Abstract**

## Background

Cystic fibrosis (CF) is a common autosomal recessive genetic disorder, affecting approximately 10,000 and 30,000 people in the UK and USA, respectively [1, 2]. Mutations of the CF transmembrane conductance regulator (CFTR) gene can lead to defects in the encoded epithelial cell apical membrane anion channel [3]. This results in defective ion transport, airway surface liquid depletion and absent or impaired mucociliary clearance [3]. Although the disorder is multi-systemic, the primary cause of morbidity and early mortality in this disease is attributable to progressive airway and lung parenchymal damage, resulting from a vicious cycle of unchecked airway infection and inflammation [4, 5].

A relatively small group of bacterial species, all of which can be readily isolated using conventional aerobic culture-based approaches, are associated with chronic lower respiratory infection in CF, including *Pseudomonas aeruginosa*, *Staphylococcus aureus*, *Burkholderia cepacia* complex, *Haemophilus influenzae*, *Stenotrophomonas maltophilia* and *Achromobacter xylosoxidans* [1]. Culture-based approaches have influenced everything from the way infections are treated to informing national CF registries on changing pathogen prevalences with age [6, 7]. However, molecular approaches have elucidated a much more complex picture of polymicrobial lower airway infection in this disease [8–10]. In light of the recognition that CF lung microbiota are multifarious, the limitations of culture-based diagnostic microbiology to characterise CF lung infections have become increasingly apparent [7]. The traditional ‘one microbe, one disease’ concept of infection pathogenesis and infection control in CF management has therefore been brought into question [6, 11, 12].

A crucial challenge in CF is how to use microbiomics to direct clinical management of airway infections. In a broader human microbiome context, it has been strongly advocated that interventions which could help treat a range of conditions, including chronic lung infections, will only be discovered by understanding the ecological and evolutionary relationships that members of a microbiota have with each other and with their host [13, 14]. A classical approach in traditional ecology has been to identify and study ecological patterns and subsequently proceed onto understanding the processes that generate those patterns [15, 16]. One potential pattern in the CF lower respiratory tract that warrants further investigation is that of a relationship between lung microbiota diversity and lung function [8, 10, 17, 18].

Forced expiratory volume in 1 s (FEV_1_), expressed as a normalised percent of the predicted value (%FEV_1_) [19], is widely used to monitor lung function and describe lung disease severity in CF and other lung diseases [20, 21]. Further, %FEV_1_ is useful as a clinical decision tool (i.e. whether to intensify treatment), as an outcome measure in clinical trials, as an important determinant in the timing of lung transplantation and as a predictor of long-term survival [22–24]. As such, %FEV_1_ is a key clinical outcome in cystic fibrosis and is currently the single best available clinical indicator of health for individuals living with the disease [1, 2, 23, 24].

The relationship of decreasing microbiota diversity with a reduction in lung function is an emergent ecological pattern in CF that has potential as an informative indicator of lung disease state in CF. However, evidence for this nascent pattern originated from microbiota studies based on small patient cohorts from single CF centres [8, 10, 17, 18]. To ascertain if this pattern is generalised requires testing with larger subject groups from multiple CF centres, encompassing the high interpatient variability inherent in CF [10, 25, 26]. In traditional ecology, it is generally anticipated that a reduction of species diversity will occur as a consequence of an environmental perturbation, such as a pollution event [27, 28]. Under these scenarios, unperturbed species-rich assemblages are typically evenly distributed but following a perturbation are replaced by species-poor-ones with high dominance and a restricted set of species [27, 28]. In a CF context, a reduction in %FEV_1_ could be taken as analogous to an environmental perturbation.

In the current study, we assessed sputum samples from a large multi-centre cohort of 299 individuals from 13 CF centres in Europe and the USA, inclusive of CF patients representing a broad cross-section of respiratory disease (Table 1). We employed high-throughput targeted amplicon sequencing to define the bacterial microbiota in the lower airways of each participant. This allowed us to determine whether the relationship between diversity and lung function holds and therefore is a generalised ecological pattern of CF lung microbiota. Further, it allowed us to ascertain if declines in lung microbiota diversity were accompanied with an increase in lung microbiota dominance. It also enabled us to elucidate the distribution of bacterial taxa, including recognised CF pathogens, across patients in relation to increasing lung disease severity. Additionally, we explored clinical and demographic factors that could explain variance in the CF lower airway microbiota.

**Table 1:** Clinical characteristics for all patients and when stratified by lung disease category

		Lung disease category^a^		
		Severe	Moderate	Mild
	All patients	< 40%	40–69%	≥ 70%
Number of patients	299	101	139	57
Sex (female:male)	137:160	39:62	66:73	32:25:00
Mean age (±SD)^b^	29.9 (± 10.2)	30.0 (± 10.8)	29.7 (± 10.1)	30.5 (± 9.5)
Mean %FEV_1_ (±SD)	49.5 (± 22.0)	25.5 (± 7.7)	53.2 (± 8.4)	82.8 (± 9.2)
CFTR Genotype^c^				
Homozygous F508del	153	46	75	32
Heterozygous F508del	105	38	47	20
Non-F508del	39	17	17	5
Clinical status (stable:exacerbation)^d^	86:211	43:58	38:101	5:52
CF related diabetes	119	54	50	15
Pancreatic insufficiency	250	91	111	48
Region				
Europe	161	58	73	30
USA	136	43	66	27
CF Centre				
Bedford, NH, USA	17	5	9	3
Belfast, Northern Ireland	27	12	13	2
Boston, MA, USA	16	6	5	5
Burlington, VT, USA	30	8	17	5
Dublin, Ireland	1	0	1	0
Lebanon, NH, USA	6	2	2	2
London, UK	6	1	5	0
New York, NY, USA^e^	5	2	1	0
Newcastle, UK	26	16	8	2
Portland, ME, USA	25	14	6	5
Seattle, WA, USA	39	6	26	7
Southampton, UK	94	29	39	26
Warsaw, Poland	7	0	7	0
Antibiotics^f^				
Amikacin	12	6	6	0
Azithromycin	64	34	22	8
Aztreonam	59	24	27	8
Ceftazidime	55	20	30	5
Ciprofloxacin	17	3	12	2
Colistin	59	32	21	6
Co-trimoxazole	13	5	5	3
Flucloxacillin	24	14	9	1
Fosfomycin	10	5	5	0
Meropenem	42	16	21	5
Tobramycin	120	49	52	19
Other antibiotics	113	39	58	14

^a^Predicted %FEV_1_ used to define lung disease categories. ^b^Age in years at time of sampling (minimum age 12 years, maximum 72 years), ^c^CFTR genotype cystic fibrosis transmembrane conductance regulator genotype. Homozygous F508del, two copies of the F508del gene mutation. Heterozygous F508del, single copy of this mutation plus another mutation. ^d^Exacerbation is protocol-defined as receiving IV antibiotic treatment for worsening pulmonary status, as determined by the treatment team. ^e^Two patient samples from this centre were excluded from further analyses due to incomplete metadata. ^f^Defined as having received a particular antibiotic within 2 weeks prior to sputum sampling. For brevity, only antibiotics administered to 10 or more of all patients are reported above. Other antibiotics included augmentin, cefepime, cefoxitin, ceftriaxone, cefuroxime, chloramphenicol, clindamycin, doripenem, doxycycline, imipenem, levofloxacin, linezolid, metronidazole, minocycline, moxifloxacin, rifampicin, tazocin, teicoplanin, temocillin, tigecycline and vancomycin

## Results

**Fig. 1:**
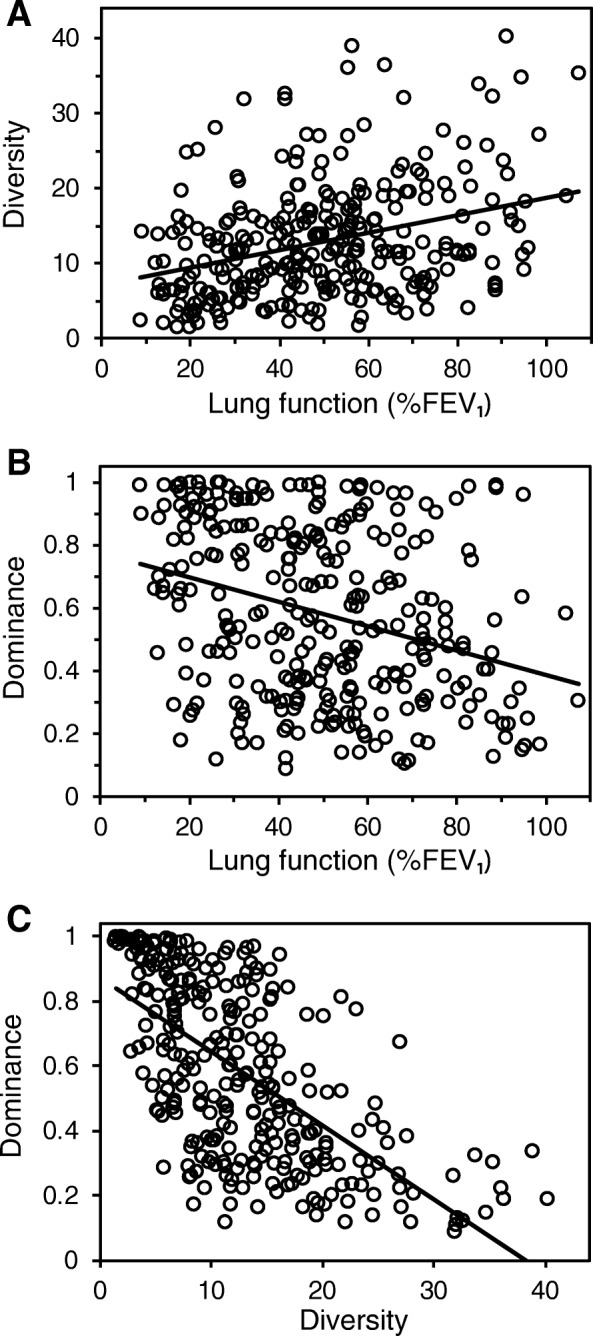
Relationships between microbiota diversity, dominance and lung function. **a** Fisher’s alpha index of diversity plotted against percent predicted forced expiratory volume in 1 s (%FEV_1_). **b** Berger-Parker dominance index and %FEV_1_. **c** Berger-Parker dominance index plotted against Fisher’s alpha index of diversity. In each case linear regression lines have been fitted: (**a**) *r*^2^ = 0.11, *F*_1,295_ = 36.7, *P* < 0.0001; (**b**) *r*^2^ = 0.10, *F*_1,295_ = 31.2, *P* < 0.0001 and (**c**) *r*^2^ = 0.41, *F*_1,295_ = 202.6, *P* < 0.0001

From 297 patient respiratory samples included in the final analyses (Table 1), 598 distinct bacterial operational taxonomic units (OTUs) were identified, with a mean (± SD) of 86.5 (± 47.3) OTUs per sample, and a minimum and maximum of 13 and 267 OTUs, respectively. Relationships between microbiota diversity and dominance with lung function were tested with linear regression (Fig. 1). Both diversity and dominance demonstrated significant linear relationships with %FEV_1_, wherein diversity decreased and dominance increased with a reduction in lung function. Further, a significant negative correlation was found between diversity and dominance, in that as diversity decreased, dominance increased (Fig. 1). In order to examine the relationships between lung function and lung microbiota characteristics further, patients were stratified into lung disease categories, as described in the US CF Foundation Patient Registry [1]. In this schema, lung function (as measured by %FEV_1_) is categorised as follows: greater than or equal to 70% predicted indicates mild/normal lung disease, 40–69% predicted indicates moderate lung disease and less than 40% predicted indicates severe lung disease [1].

**Fig. 2:**
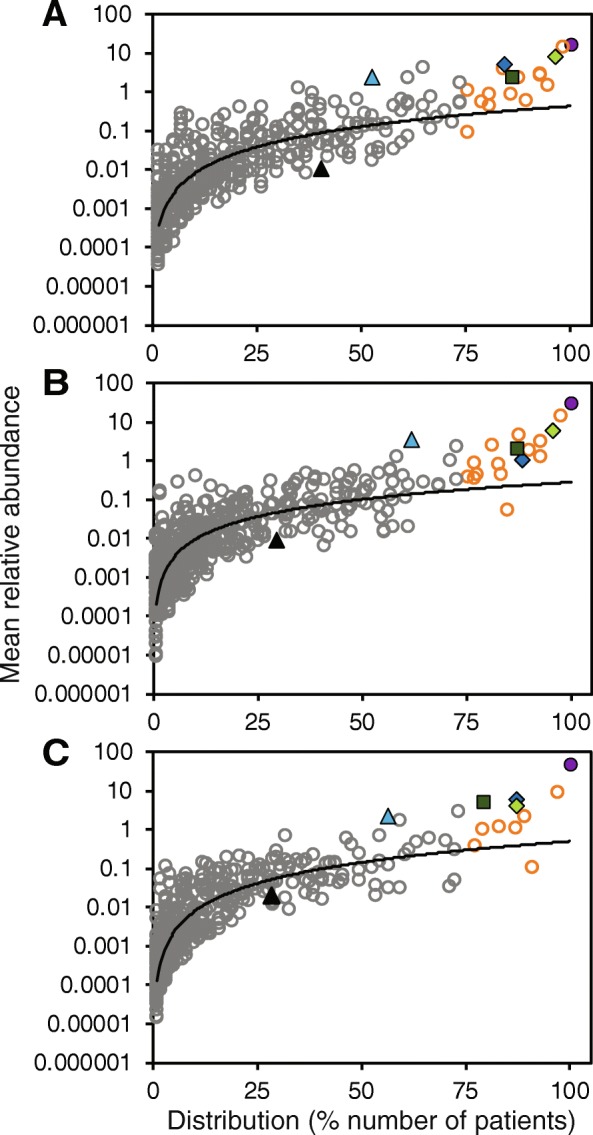
Distribution and abundance of bacterial taxa across patients in worsening lung disease categories. **a** Mild/normal. **b** Moderate. **c** Severe categories. Given is the percentage number of patient respiratory samples each bacterial taxon was observed to be distributed across, plotted against the mean percentage abundance across those samples. Core taxa are defined as those that fall within the upper quartile of distribution (orange circles), and satellite taxa (grey circles) defined as those that do not. Recognised pathogens are marked as follows: *Pseudomonas aeruginosa*, purple circle; *Staphylococcus aureus*, light green diamond; *Stenotrophomonas maltophilia*, blue diamond; *Burkholderia cepacia* complex, green square; *Haemophilus influenzae*, light blue triangle and *Achromobacter xylosoxidans*, black triangle. Distribution-abundance relationship regression statistics: (**a**) *r*^2^ = 0.64, *F*_1,514_ = 927.3, *P* < 0.0001; (**b**) *r*^2^ = 0.62, *F*_1,581_ = 961.9, *P* < 0.0001; (**c**) *r*^2^ = 0.75, *F*_1,527_ = 1549.1, *P* < 0.0001. Common taxa are listed Table S1

Bacterial taxa were partitioned into either common and abundant core taxa or rarer and infrequent satellite taxa, based upon their prevalence and relative abundance across samples within each lung disease category (Fig. 2). Within the mild/normal category, 17 core and 499 satellite taxa occurred, with the former accounting for 64.1% of the cumulative relative abundance. In the moderate category, 17 core taxa accounting for 71.8% of the abundance, and 566 satellite taxa occurred. Within the severe category, in addition to 518 satellite taxa, 11 core taxa with a cumulative abundance of 78.7% occurred. Further, core or satellite status of recognised CF pathogens was determined. Within each lung disease category, four OTUs corresponding to recognised CF pathogens, *P. aeruginosa*, *S. aureus*, *S. maltophilia* and *B. cepacia* complex, had core status, while two, *H. influenzae* and *A. xylosoxidans*, were satellite taxa (Fig. 2). Core taxa for each lung disease category are given in Table S1.

**Fig. 3:**
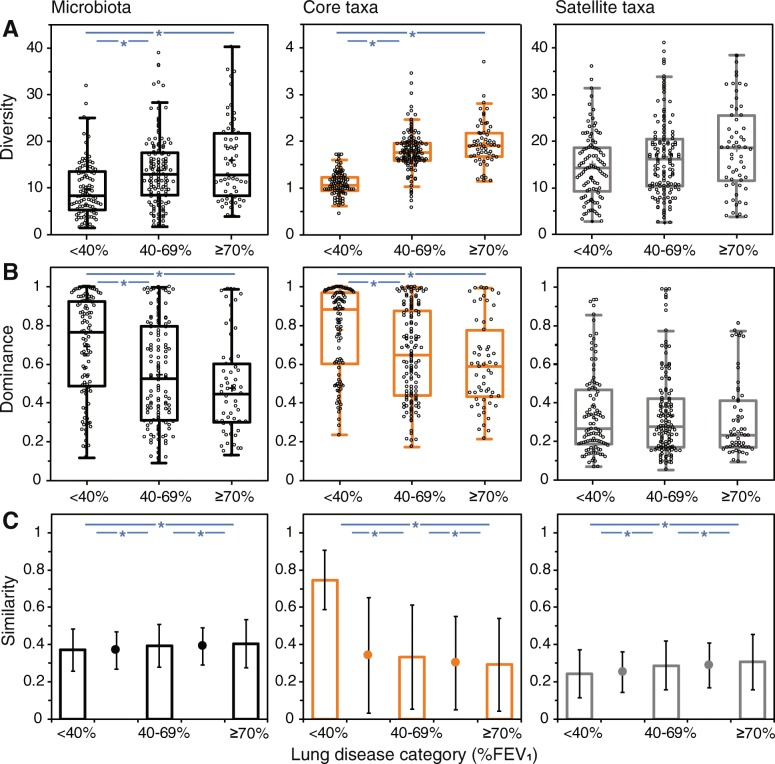
Comparison of microbiota diversity, dominance and composition when stratified by lung disease category. In each instance, relationships within the microbiota, core taxa and satellite taxa are given. Changes in (**a**) Fisher’s alpha index of diversity and (**b**) Berger-Parker dominance index with lung disease category (%FEV_1_). Boxplots show 25–75th interquartile (IQR) range with whiskers showing 1.5 times IQR. Black circles indicate individual patients and cross symbol represents the mean. Asterisks denote significant differences in diversity or dominance between two lung disease categories following both Kruskal-Wallis tests and Hedges’ *d* effect size analysis.(**c**) Variation in microbiota composition within (columns) and between (circles) lung disease categories using the Bray-Curtis index of similarity. Error bars represent standard deviation of the mean. Asterisks denote significant differences in composition between lung disease categories following one-way PERMANOVA tests with Bonferroni correction. Summary statistics for Kruskal-Wallis and PERMANOVA analyses are provided in supplementary Tables S2, S3 and S4. Hedges’ *d* effect size analyses are provided in Figure S1

Common patterns of decreasing diversity with increasing lung disease severity were observed for the microbiota, the core taxa and satellite taxa (Fig. 3a). Kruskal-Wallis tests and Hedges’ *d* effect size measures were used to determine whether Fisher’s alpha indices of diversity were significantly different between lung disease categories (Fig. 3a, Table S2 and Figure S1). Diversity was significantly lower in the severe category when compared to the moderate and mild/normal categories in the microbiota and core taxa. Conversely, the opposite pattern was observed for dominance within the microbiota and core taxa group, where dominance was significantly higher in the severe category when compared to the two other categories, as determined by Kruskal-Wallis tests and Hedges’ *d* effect size measures (Fig. 3b; Table S3 and Figure S1). No significant relationships between diversity or dominance and disease category were found in the satellite taxa group.

**Fig. 4:**
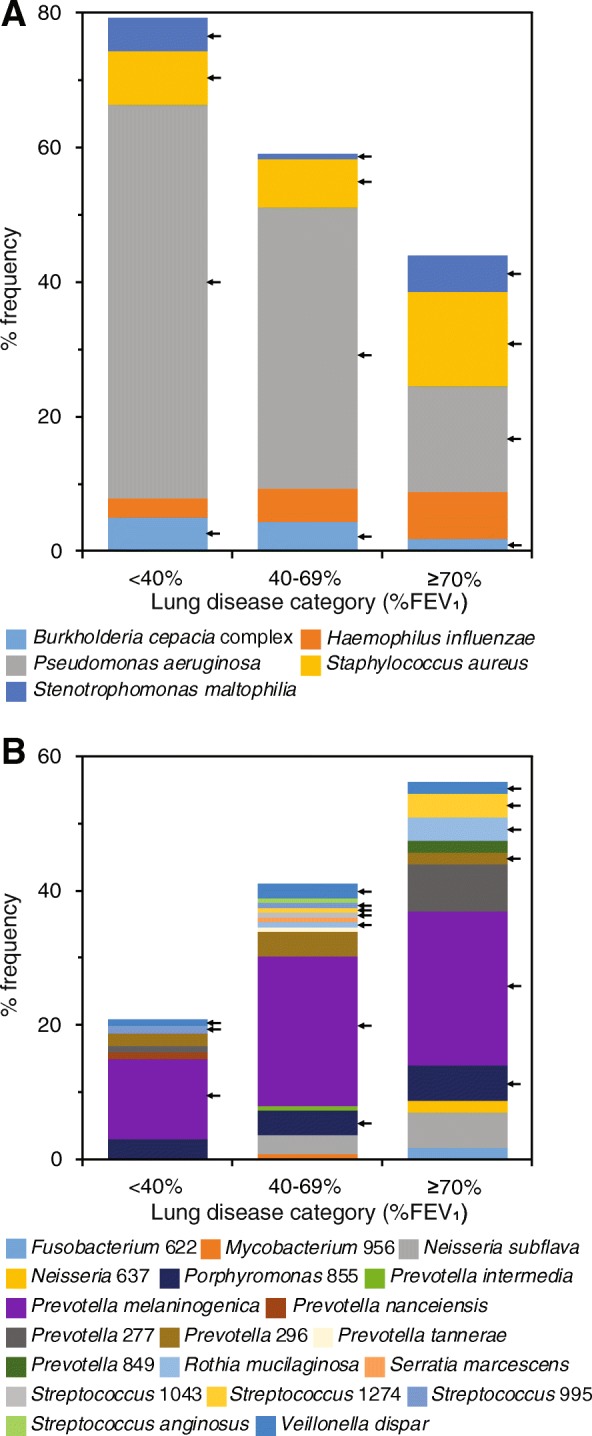
Dominant bacterial taxa across lung disease categories. Percent frequency of dominance for (**a**) recognised CF pathogens and (**b**) other bacterial taxa in each lung disease category. Dominant taxon is defined as the most abundant taxon by relative abundance within a given lung microbiota sample

Permutational multivariate analysis of variance (PERMANOVA) tests determined that the compositions of the microbiota, the core taxa and satellite taxa were significantly different across the strata of lung disease (Fig. 3c, Table S4). For the core taxa, within category similarity notably increased with decreasing lung function, ranging from a mean Bray-Curtis similarity (±SD) of 0.29 ± 0.25 in the mild/normal category to 0.75 ± 0.16 in the severe category (Fig. 3c, Table S4). Similarity of percentages (SIMPER) analysis allowed determination of which taxa contributed most to the dissimilarity in microbiota composition across the lung disease categories (Table 2). From the top six OTUs that contributed most to the dissimilarity, these included five identified as recognised CF respiratory pathogens, including *P. aeruginosa*, *S. aureus*, *B. cepacia* complex, *S. maltophilia* (all core taxa in all categories) and *H. influenzae* (satellite taxon in all categories). Additionally, the second top taxon was an OTU identified as belonging to the *Prevotella* genus, putatively labelled as *P*. *melaninogenica*. The remaining taxa within the SIMPER table predominantly comprised OTUs from the *Streptococcus* genus or OTUs from genera consisted of strict anaerobic species, including *Prevotella*, *Porphyromonas, Rothia* and *Veillonella* (Table 2). As a complement to the SIMPER analysis, the frequency of which taxa dominated patient’s lower airway microbiota within and across lung disease categories was determined (Fig. 4). A clear pattern emerged of increasing dominance by recognised pathogens, which was mainly driven by the OTU identified as *P. aeruginosa*, as lung function decreased (Fig. 4a). Conversely, better lung function associated with increasing dominance by other bacterial taxa, especially the putative *P. melaninogenica* OTU (Fig. 4b).

**Table 2:**
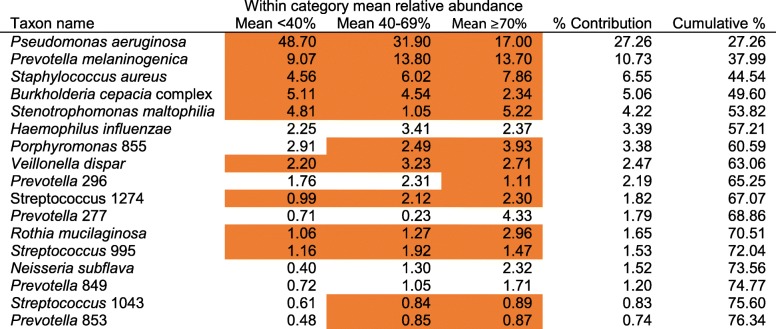
Similarity of percentage (SIMPER) analysis of microbiota dissimilarity (Bray-Curtis) between lung disease categories

Core taxa in a given lung disease category are highlighted in orange. Also given is within category mean percent abundance for taxa. Percentage contribution is the mean contribution divided by mean dissimilarity across samples (62.3%). The list of species is not exhaustive, so cumulative percent does not sum to 100%. Operational taxonomic unit (OTU) identifications have been used for bacterial taxon names. OTU numbers have been used to differentiate between taxa within the same genus. Given the length of the ribosomal sequences analysed, species identities should be considered putative

Redundancy analysis (RDA) was used to relate the variability in the composition of the lung microbiota, the core taxa and satellite taxa to clinical/demographic factors (outlined in Table 1) and geographical distance between CF centres. Principal coordinates of neighbour matrices (PCNM) were calculated from grid coordinates of the 13 CF centres and used as explanatory spatial variables for RDA. Based on the RDA direct ordination approach, the microbiota, core taxa and satellite taxa were significantly correlated with factors listed in Table 3. Antibiotic exposure and %FEV_1_ were the most significant factors in explaining variance within the microbiota and core taxa, followed to a lesser extent by patient age and region in which a patient’s CF centre was located (i.e. Europe or USA, Table 1). For the satellite taxa, again antibiotic exposure was the most significant factor along with, albeit to a lesser extent, %FEV_1_ (Table 3). Other significant clinical/demographic factors included patient age, patient sex, clinical status, CFTR genotype and geographic region. Notably, geographical distance between CF centres was a significant factor only for the satellite taxa, accounted for by three of six PCNM vectors.

**Table 3:** Redundancy analyses for determination of percent variation in the lung microbiota, core taxa and satellite taxa explained by significant clinical and geographical distance variables between centres

	Microbiota			Core taxa			Satellite taxa		
	Var. Exp (%)	pseudo-*F*	*P* (adj)	Var. Exp (%)	pseudo-*F*	*P* (adj)	Var. Exp (%)	pseudo-*F*	*P* (adj)
Age	2.2	3.6	0.046	2.4	4	0.021	1.4	2.1	0.008
Antibiotics									
Aztreonam							1.4	2.1	0.013
Ceftazidime							1.4	2	0.008
Colistin	2.2	3.6	0.036	2.2	3.2	0.042	1.2	1.8	0.008
Flucloxacillin	2.4	3.7	0.042	5.4	8.2	0.016	1.2	1.8	0.007
Meropenem	4.4	6.6	0.028	2.4	3.6	0.033	2.4	3.5	0.021
Tobramycin	1.8	2.9	0.040	2.2	3.2	0.032	1.2	1.9	0.007
Clinical status							1	1.4	0.022
%FEV_1_	10.2	16.3	0.037	10.4	17.1	0.027	2.5	3	0.014
CFTR genotype							1	1.6	0.007
Sex							1.2	1.9	0.025
Region	1.8	3	0.049	2	3.3	0.041	1.4	2.1	0.009
Distance									
PCNM1							1.4	2.1	0.006
PCNM3							1	1.4	0.036
PCNM5							1.4	2.1	0.006
Clinical	25.0			27.0			17.3		
Distance							3.8		
Total	25.0			27.0			21.1		

Principle coordinates neighbour matrices (PCNM) were used as potential explanatory values of distance between CF centres. *Var. Exp* (*%*) the percentage of microbiota variation explained by a parameter given the redundancy analysis. *P*(*adj*) adjusted significance value following false discovery rate correction. Clinical status is stable or in treatment for acute pulmonary exacerbation

## Discussion

Chronic infection of the lower airways is undeniably polymicrobial, e.g. [8–10, 25, 26, 29], and remains the leading cause of morbidity and mortality for those living with CF [1–3]. However, current infection surveillance and infection control approaches in CF remain constrained by classical aerobic culture-based diagnostic microbiology; screening only for the presence or absence of a limited palette of targeted bacterial species [1, 2]. The unanswered question of how to translate a more complete understanding of the lower airway microbiota, which typically consists of bacterial taxa ranging from strict aerobes to obligate anaerobes, to novel treatment strategies, is a major reason why microbiome analysis is not yet employed in the clinical arena.

A pivotal step toward realising the full potential of microbiota information in the management of lower airway infection in CF is to understand the ecology of the lung microbiome [10, 13, 14], and identify ecological patterns of microbiota diversity in the disease as it progresses [15, 16]. Studies that either incorporate large cross-sectional cohorts from multiple CF centres and encompassing the high interpatient variability inherent in CF or in-depth longitudinal studies, which provide increased statistical power and clearer insight for further investigation, are therefore required. Using the former approach, we tested and confirmed a significant relationship between decreasing microbiota diversity and reduced lung function (Fig. 1). As such, that relationship can be considered as a generalised ecological pattern of CF microbiota (Fig. 1). Moreover, the loss of diversity was accompanied by an increase in dominance, which would also be a broader expected outcome when communities face environmental perturbations in ecological studies [27, 28]. When the pattern between lung function and diversity was observed as part of previous small cohort/single centre studies, it was characterised in each instance with low coefficient of determination values [8, 10, 17, 18]. This was also the case in the current study, and we posit that this results from high interpatient variability (Fig. 1) [10, 25, 26]. Subsequently, we stratified patients into lung disease categories, of increasing disease severity, to investigate further the relationships between microbiota characteristics and lung function, and the factors contributing to the variance in the microbiota.

We have previously established that the categorisation of microbiota into core and satellite taxa reveals important aspects of metacommunity species-abundance distributions that would be neglected without such a distinction [10, 30, 31]. A coherent metacommunity could be expected to exhibit a direct positive relationship between the prevalence and relative abundance of individual taxa across constituent communities [28]. Consistent with this prediction, the proportional abundance of bacterial OTUs in each lung disease category significantly correlated with the number of individual sample communities those taxa occupied (Fig. 2). Additionally, it should be expected that the core taxa would account for the majority of relative abundance and the rarer satellite taxa account for the majority of the diversity within a metacommunity [10, 30, 31]. This was the case in the current study, where the core taxa increasingly accounted for greater total relative abundance with increasing disease severity. Moreover, the high variability observed in microbiota diversity was reflected in the satellite taxa, but not in the core, indicating that the rarer taxa underpinned the observed variance in overall diversity (Fig. 3a). Conversely, increasing microbiota dominance patterns were mirrored by the abundant and prevalent core taxa (Fig. 3b), and core taxa composition was especially conserved in the severe category when compared to the other categories (Fig. 3c). In summary, changes in CF airway microbiota diversity and dominance follow predictions of the ecological theory, and that composition becomes more conserved with increasing selective pressure from harsher perturbations [27, 32]. In a CF context, the selective pressure on microbiota composition associated with worsening lung function may result from increased inflammation and intensified antibiotic therapy to treat chronic infection and recurrent exacerbations [22–24].

In general, it is understood that the common and prevalent core taxa contribute significantly to ecosystem function, carrying out the majority of functional activity, while the rare and infrequent satellite taxa can represent the influence of immigration and seedbank of diversity that can thrive and dominate when conditions change [10, 33]. If we consider bacterial pathogenesis as an ecological, albeit undesirable, function within the CF lung microbiome, then one would predict that recognised CF pathogens would be members of the abundant and prevalent core taxa, would contribute heavily to microbiota compositional similarity and would dominate the lung microbiota of many individual patients.

We found that this was not universally the case across our study group (Fig. 2 and Table S1). Derived from presence/absence culture screening data, *P*. *aeruginosa* and *S*. *aureus* are reported and recognised as dominant pathogens of concern in CF based on their prevalence [1, 34]. That was reflected here in terms of both the prevalence and relative abundance of the corresponding OTUs for those pathogens (Fig. 2 and Table S1). Conversely, *B. cepacia* complex, *S. maltophilia*, *A. xylosoxidan, and Haemophilus influenzae* are reported as being less prevalent, with culture positive reporting in < 20% of USA CF patients [1]. Here, OTUs identified as those pathogens all had greater prevalences than culture-based data, with *B. cepacia* complex and *S. maltophilia* found to be core taxa (Fig. 2 and Table S1). A probable reason for the higher prevalences is the increased sensitivity inherent in molecular-based approaches when compared to culture-based methods [7]. SIMPER analysis revealed that all recognised pathogen OTUs, with the exception of *A. xylosoxidans*, contributed substantially to the dissimilarity between lung disease categories (Table 2). In addition, the lung microbiota of individual patients became increasingly dominated by recognised pathogen OTUs, and especially by the *P. aeruginosa* OTU, in concert with decreasing lung function (Fig. 4). Again, *A. xylosoxidans* stood as an exception to this rule. Our findings, therefore, bring into question the perceived importance of this species in CF.

Conversely, other bacteria, but especially OTUs identified as belonging to genera comprised of obligate anaerobes, were observed to increasingly dominate microbiota of patients with better lung function (Fig. 4). Taxa belonging to the genera of *Prevotella*, *Porphyromonas*,and *Veillonella*, as observed here, have been previously associated with better clinical outcomes when they dominate lung microbiota [35]. Although defective mucociliary clearance in CF make it difficult to eradicate pathogenic bacteria, it might be possible to mitigate the effects of resident pathogens by promoting growth of bacterial taxa whose dominance is associated with better outcomes [11]. Reproducible infection models, such as CF specific air liquid interface cell cultures, might be used to identify paradigms to manage microbiota community structure [36]. Further, combining these paradigms with longitudinal patient studies might elucidate the underlying mechanisms that govern microbial diversity and dominance in the CF lung, and the role played by intensive antibiotic administration in the context of advancing lung disease [11].

While we established unambiguous relationships between lung microbiota characteristics (diversity, dominance and composition) and lung function, other clinical factors appear to contribute to the observed high interpatient variation. In particular, antibiotic exposure significantly explained variation in the composition of the microbiota and the core and satellite taxa groups (Table 3). This is unsurprising as most CF patients are throughout their lives frequently on some form of antibiotic treatment, ranging from eradication to chronic suppressive therapies [3, 34]. Here, all of the specific antibiotics that were significant in explaining variation in microbiota composition are administered to target specific recognised pathogens [34].

To a lesser extent, patient age and region (Europe or USA) also explained microbiota variance across the core and satellite taxa, and the whole microbiota (Table 3). Age has previously been found to weakly associate with microbiota characteristics, with fluctuations in diversity mainly happening in childhood [25, 26]. With regard to region, a possible explanation for the effect could relate to patient characteristics, which can vary according to country of treatment [37]. However, biogeographical influences may also be at play, with the local environment acting as a source of immigration for bacterial taxa found in a patient’s lower airways [37, 38]. Here we tested whether the geographical distance between participating CF centres significantly correlated with microbiota composition (Table 3). This questioned the biogeographical assumption that patients attending centres that are closer together have more similar microbiota than those that are further apart [38]. We found that this was not the case for the core taxa, but did significantly explain variation in the satellite taxa group which, as noted earlier, represents the influence of immigration in a community [33]. Interestingly, clinical status, defined as whether a patient was receiving treatment for pulmonary exacerbation or was judged clinically stable, was a significant factor for explaining variation in the satellite taxa but not the core taxa (or microbiota). This agrees with our previous work, which revealed core and satellite group compositions were resistant and resilient, respectively, to pulmonary exacerbation and antibiotics interventions [30]. Though not incorporated in the current study, measures of inflammatory markers and immune response could certainly account for variation within the infection microbiota and should be integrated into future studies of host-microbiota interactions in CF [35].

## Conclusions

Establishing how best to utilise microbiota information in CF infection management offers great promise to further improve the lives of people living with CF. Translating the complexity of the lower airway microbiota into simplified yet clinically interpretable ecological metrics is a pragmatic way forward. Our findings, from a cohort of CF patients spanning a wide spectrum of lung disease and from different geographic regions indicate that microbiota diversity and dominance (as well as the identity of the dominant bacterial species), in combination with lung function measures (%FEV_1_), can be used as informative indicators of disease state. A recent study that focused on early end-stage lung disease (eESLD) in CF supports this view [39]; where eESLD patients were more likely to have low microbiota diversity dominated by specific recognised pathogens, including *P. aeruginosa*. More broadly, and given the high interpatient variability inherent in CF and found in this study, we recommend that microbiota sampling become part of routine microbial surveillance in the same manner that culture-based approaches are currently employed. This longitudinal surveillance of individual patients in a given CF centre would refine monitoring of changes in microbiota characteristics and lung function, and potentially improve personalised treatment of the disease.

## Methods

### Study design and subjects

Spontaneously expectorated sputum samples were provided from 299 adolescent to adult individuals with CF (one sample per patient), representing a broad cross-section CF respiratory disease, attending 13 CF centres in Europe and the USA (Table 1). The study was approved by either local research ethics committee (UK) or institutional review board (USA) (see Ethics approval and consent to participate section below). Each centre collected demographic and medical data on participating patients, including information on age, lung function, antibiotic use and other data (summarised in Table 1). All samples were stabilised at – 80 °C within 12 h of collection and freeze-thawing of samples kept within 3 cycles, to reduce introduction of bias as previously described [40, 41]. Two samples (COL0003 and COL0005) were excluded from the main analyses due to missing metadata, including %FEV_1_. Metadata is available at figshare.com under 10.6084/m9.figshare.9848513.v1.

### Targeted amplicon sequencing

Sputum samples were washed three times with 1X phosphate-buffered saline to remove saliva, to reduce potential bias from upper airway microbiota, as previously described [42]. DNA from dead or damaged cells, as well as extracellular DNA (which could bias final sequence analysis) was excluded from analysis via cross-linking with propidium monoazide prior to DNA extraction, as previously described [43]. Approximately 50 ng of template DNA was amplified using Q5**®** high-fidelity DNA polymerase (New England Biolabs, Hitchin, UK), each with a unique dual-index barcode primer combination [44]. Individual PCR reactions employed 25 cycles of an initial 30 s, 98 °C denaturation step, followed by annealing phase for 30 s at 50 °C and final extension step lasting 60 s at 72 °C. Primers were based upon the universal primer sequence 27F and 338R [44]. An amplicon library consisting of ~ 300 bp amplicons spanning the V1-V2 hypervariable regions of the 16S rRNA gene was sequenced on the Illumina MiSeq platform using V3 chemistry at the Wellcome Sanger Institute, Cambridgeshire, UK. Mock communities, DNA extract and PCR negative controls were included in each sequencing run [45].

### Sequence analysis

Sequenced paired-end reads were joined using PEAR [46], quality filtered using FASTX tools (http://hannonlab.cshl.edu). Chimeras were identified and removed with VSEARCH_UCHIME_REF [47] using Greengenes Release 13_5 [48]. Singletons were removed and the resulting sequences were clustered into operational taxonomic units (OTUs) at 97% sequence identity using VSEARCH_CLUSTER_FAST. Representative sequences were taxonomically assigned by RDP Classifier with the bootstrap threshold of 0.8 or greater using Greengenes Release 13_5 as a reference [48]. The raw sequence data reported in this study have been deposited in the European Nucleotide Archive under study accession number PRJEB30646. From the 297 samples used, a total of 5,752,628 bacterial sequence reads (mean ± standard deviation per sample, 19,240 ± 17,233) were included in the final analysis, identifying 598 distinct bacterial OTUs to genus/species level. Given the length of the ribosomal sequences analysed, these identities should be considered putative.

### Statistical analysis

Regression analysis, coefficients of determination (*r*^2^), degrees of freedom (df), *F*-statistic and significance (*P*) were calculated using XLSTAT v2018.1 (Addinsoft, Paris, France). Fisher’s alpha index of diversity was calculated in PAST v3.20 (http://folk.uio.no/ohammer/past). This measure of diversity is relatively unaffected by variation in sample size, and completely independent if sequence reads per sample > 1000 [28]. The Berger-Parker index of dominance was calculated in PAST. This index is a measure of the numerical importance of the most abundant taxon in a given microbiota sample [28].

Recognised CF pathogens were those defined in the CF Foundation Patient Registry reporting [1]. Patients samples were stratified into lung disease categories following %FEV_1_ predicted classifications used in the CF Foundation Patient Registry reporting (mild/normal, %FEV_1_ ≥ 70%; moderate, 40–69% and severe, < 40%) [1]. Within each lung disease category, bacterial taxa were partitioned into core and satellite taxa groups, as previously described [31]. Based on a significant positive distribution-abundance relationship, the prevalent and abundant core taxa were defined as those present in more than 75% of samples, while taxa falling outside of the upper quartile were considered as satellite [30, 31].

Significant differences in diversity and dominance between groups were determined using Kruskal-Wallis analysis in conjunction with the post hoc Dunn test, and performed in XLSTAT. Additionally, effect sizes based on the comparisons of diversity or dominance were performed using Hedges’ *d* effect size measures, as described previously [43]. Sequence read data was percentage normalised for subsequent microbiota compositional-based analyses. The Bray-Curtis quantitative index of similarity was used for measures of microbiota compositional similarity throughout [28]. Permutational multivariate analysis of variance (PERMANOVA) with Bonferroni correction was used to test for significance in microbiota composition and performed in PAST. Similarity of percentages (SIMPER) analysis, to determine which taxa contributed most to compositional differences between groups, was performed in PAST. Direct ordination, by means of redundancy analysis (RDA), was used to relate variability in microbiota composition to clinical and demographic factors (Table 1) and geographical distance between CF centres. Principle coordinates of neighbour matrices (PCNM) were used as explanatory spatial variables [38] and were calculated from grid coordinates of the sites using GUSTA ME [49]. RDA was performed in CANOCO v5 [50]. Clinical/demographic variables and PCNM that significantly explained variation were determined with forward selection (999 Monte Carlo permutations with false discovery rate) and used in RDA [51]. Partial RDA was performed when both PCNM and clinical/demographic factors were significant to summarise the part of the microbiota variation explained by clinical/demographic factors after controlling the effects of geographic distance (PCNM) [51].

## Supplementary information

**Additional file 1: Table S1.** Core taxa within each lung disease category. Given is prevalence, the number of samples a given core taxon was detected in, and average relative abundance across those samples. Operational taxonomic unit (OTU) identifications have been used for bacterial taxon names. OTU numbers have been used to differentiate between taxa within the same genus. Given the length of the ribosomal sequences analysed, species identities should be considered putative.

**Additional file 2: Table S2.** Kruskal-Wallis summary statistics for testing for significant differences in diversity between lung function categories. Given for each test is the mean Fisher's alpha diversity index, standard deviation of the mean, *H*-statistic, and significance (*P*), and mean of ranks values. Asterisks denote significant differences in diversity following Kruskal-Wallis with *post-hoc* Dunn test.

**Additional file 3: Table S3.** Kruskal-Wallis summary statistics for testing for significant differences in diversity between lung function categories. Given for each test is the mean Berger-Parker index of dominance, standard deviation of the mean, *H*-statistic, and significance (*P*), and mean of ranks values. Asterisks denote significant differences in diversity following Kruskal-Wallis with *post-hoc* Dunn test.

**Additional file 4: Figure S1.** Measures of Hedges’ *d* effect size based on comparisons of (A) diversity and (B) dominance in the microbiota, core taxa, and satellite taxa, when stratified into lung disease categories. Columns represent the effect size and error bars represent the standard error of effect size. Standard error bars that cross zero indicate no significant effect on diversity or dominance between lung disease categories. In each instance, within (A) positive effect sizes represent higher diversity in the second of the two lung disease categories being compared. Within (B) negative effect sizes represent lower dominance in the 2^nd^ of the two lung disease categories being compared. Measures of diversity and dominance when stratified by lung disease category are presented in Fig. 3a and b, respectively.

**Additional file 5: Table S4.** PERMANOVA summary statistics from testing for significant differences in microbiota composition between lung function categories. Given in each instance are mean Bray-Curtis similarity within and between categories (± standard deviation of the mean), *F*-statistic, and significance (*P*). Asterisks denote significant differences in composition following one-way PERMANOVA tests with Bonferroni correction.

## Data Availability

The raw sequence data reported in this study have been deposited in the European Nucleotide Archive under study accession number PRJEB30646. Clinical and demographic metadata has been deposited at figshare.com under 10.6084/m9.figshare.9848513.v1.

## Abbreviations

CF: Cystic fibrosis
CFTR: Cystic fibrosis transmembrane conductance regulator
%FEV_1_: Percent predicted forced expiratory volume in one second
OTU: Operational taxonomic unit
SD: Standard deviation of the mean
PERMANOVA: Permutational multivariate analysis of variance
SIMPER: Similarity of percentages
PCNM: Principle coordinates of neighbour matrices
RDA: Redundancy analysis
eESLD: Early end-stage lung disease
