# Supplementary material for: Lung function and microbiota diversity in cystic fibrosis
Source: Microbiome. 2020 Apr 2;8:45. doi: 10.1186/s40168-020-00810-3 (PMC7114784; doi:10.1186/s40168-020-00810-3)
Supplement: Supplementary file 2 — Additional file 1: Table S1. Core taxa within each lung disease category. Given is prevalence, the number of samples a given core taxon was detected in, and average relative abundance across those samples. Operational taxonomic unit (OTU) identifications have been used for bacterial taxon names. OTU numbers have been used to differentiate between taxa within the same genus. Given the length of the ribosomal sequences analysed, species identities should be considered putative. [file 40168_2020_810_MOESM1_ESM.docx]

**Table S1** Core taxa within each lung disease category**.** Given is prevalence, the number of samples a given core taxon was detected in, and average relative abundance across those samples. Operational taxonomic unit (OTU) identifications have been used for bacterial taxon names. OTU numbers have been used to differentiate between taxa within the same genus. Given the length of the ribosomal sequences analysed, species identities should be considered putative.

|  | <40% | | 40-69% | | ≥70% | |
| --- | --- | --- | --- | --- | --- | --- |
|  | Prevalence | Abundance | Prevalence | Abundance | Prevalence | Abundance |
| *Pseudomonas aeruginosa* | 100 | 48.7 | 139 | 31.9 | 57 | 17.0 |
| *Burkholderia cepacia* complex | 79.2 | 5.1 | 122 | 4.5 | 49 | 2.3 |
| *Staphylococcus aureus* | 87.1 | 4.6 | 133 | 6.0 | 55 | 7.9 |
| *Porphyromonas* 855 | 0 | 0 | 113 | 2.5 | 48 | 3.9 |
| *Prevotella melaninogenica* | 97.0 | 9.1 | 136 | 13.8 | 56 | 13.7 |
| *Prevotella* 296 | 0 | 0 | 0 | 0 | 43 | 1.1 |
| *Stenotrophomonas maltophilia* | 87.1 | 4.8 | 123 | 1.0 | 48 | 5.2 |
| *Veillonella dispar* | 89.1 | 2.2 | 129 | 3.2 | 53 | 2.7 |
| *Streptococcus* 995 | 83.2 | 1.2 | 125 | 1.9 | 54 | 1.5 |
| *Streptococcus* 1274 | 79.2 | 1.0 | 121 | 2.1 | 50 | 2.3 |
| *Streptococcus* 1043 | 0 | 0 | 115 | 0.8 | 49 | 0.9 |
| *Rothia mucilaginosa* | 87.1 | 1.1 | 129 | 1.3 | 53 | 3.0 |
| *Prevotella* 853 | 0 | 0 | 107 | 0.9 | 46 | 0.9 |
| *Granulicatella* 1232 | 0 | 0 | 107 | 0.4 | 46 | 0.4 |
| *Rothia dentocariosa* | 77.2 | 0.4 | 116 | 0.4 | 51 | 0.6 |
| *Streptococcus* 1049 | 0 | 0 | 105 | 0.4 | 0 | 0 |
| *Veillonella parvula* | 0 | 0 | 108 | 0.5 | 45 | 0.6 |
| *Microbacterium* 182 | 91.1 | 0.1 | 118 | 0.1 | 43 | 0.1 |
