# Supplementary material for: Lung function and microbiota diversity in cystic fibrosis
Source: Microbiome. 2020 Apr 2;8:45. doi: 10.1186/s40168-020-00810-3 (PMC7114784; doi:10.1186/s40168-020-00810-3)
Supplement: Supplementary file 3 — Additional file 2: Table S2. Kruskal-Wallis summary statistics for testing for significant differences in diversity between lung function categories. Given for each test is the mean Fisher's alpha diversity index, standard deviation of the mean, H-statistic, and significance (P), and mean of ranks values. Asterisks denote significant differences in diversity following Kruskal-Wallis with post-hoc Dunn test. [file 40168_2020_810_MOESM2_ESM.docx]

**Table S2** Kruskal-Wallis summary statistics for testing for significant differences in diversity between lung function categories. Given for each test is the mean Fisher's alpha diversity index, standard deviation of the mean, *H*-statistic, and significance (*P*), and mean of ranks values. Asterisks denote significant differences in diversity following Kruskal-Wallis with *post-hoc* Dunn test.

|  | Test |  | Category 1 | | Category 2 | |  |  | Mean of ranks | |
| --- | --- | --- | --- | --- | --- | --- | --- | --- | --- | --- |
|  | Category 1 | Category 2 | Mean | ±SD | Mean | ±SD | *H* | *P* | Category 1 | Category 2 |
| Microbiota | <40% | 40-69% | 9.80 | 5.95 | 13.76 | 7.48 | 19.23 | <0.0001* | 137.25 | 97.45 |
|  | 40-69% | ≥70% | 13.76 | 7.48 | 15.90 | 8.90 | 1.74 | 0.187 | 106.85 | 95.08 |
|  | <40% | ≥70% | 9.80 | 5.95 | 15.90 | 8.90 | 19.46 | <0.0001* | 100.88 | 67.44 |
| Core taxa | <40% | 40-69% | 1.08 | 0.25 | 1.78 | 0.43 | 126.90 | <0.0001* | 163.50 | 61.28 |
|  | 40-69% | ≥70% | 1.78 | 0.43 | 1.96 | 0.47 | 6.24 | 0.012* | 114.31 | 92.02 |
|  | <40% | ≥70% | 1.08 | 0.25 | 1.96 | 0.47 | 97.97 | <0.0001* | 126.72 | 52.85 |
| Satellite taxa | <40% | 40-69% | 15.06 | 8.44 | 16.72 | 8.59 | 2.26 | 0.132 | 126.25 | 112.60 |
|  | 40-69% | ≥70% | 16.72 | 8.59 | 18.41 | 9.33 | 1.54 | 0.215 | 106.34 | 95.28 |
|  | <40% | ≥70% | 15.06 | 8.44 | 18.41 | 9.33 | 5.17 | 0.023* | 90.52 | 73.28 |
