# Supplementary material for: Lung function and microbiota diversity in cystic fibrosis
Source: Microbiome. 2020 Apr 2;8:45. doi: 10.1186/s40168-020-00810-3 (PMC7114784; doi:10.1186/s40168-020-00810-3)
Supplement: Supplementary file 4 — Additional file 3: Table S3. Kruskal-Wallis summary statistics for testing for significant differences in diversity between lung function categories. Given for each test is the mean Berger-Parker index of dominance, standard deviation of the mean, H-statistic, and significance (P), and mean of ranks values. Asterisks denote significant differences in diversity following Kruskal-Wallis with post-hoc Dunn test. [file 40168_2020_810_MOESM3_ESM.docx]

**Table S3** Kruskal-Wallis summary statistics for testing for significant differences in diversity between lung function categories. Given for each test is the mean Berger-Parker index of dominance, standard deviation of the mean, *H*-statistic, and significance (*P*), and mean of ranks values. Asterisks denote significant differences in diversity following Kruskal-Wallis with *post-hoc* Dunn test.

|  | Test |  | Category 1 | | Category 2 | |  |  | Mean of ranks | |
| --- | --- | --- | --- | --- | --- | --- | --- | --- | --- | --- |
|  | Category 1 | Category 2 | Mean | ±SD | Mean | ±SD | *H* | *P* | Category 1 | Category 2 |
| Microbiota | <40% | 40-69% | 0.69 | 0.26 | 0.55 | 0.27 | 16.81 | <0.0001* | 142.05 | 104.84 |
|  | 40-69% | ≥70% | 0.55 | 0.27 | 0.48 | 0.25 | 2.62 | 0.105 | 102.70 | 88.254 |
|  | <40% | ≥70% | 0.69 | 0.26 | 0.48 | 0.25 | 21.26 | <0.0001* | 92.11 | 57.19 |
| Core taxa | <40% | 40-69% | 0.78 | 0.22 | 0.65 | 0.24 | 18.98 | <0.0001* | 143.40 | 103.86 |
|  | 40-69% | ≥70% | 0.65 | 0.24 | 0.60 | 0.23 | 1.66 | 0.198 | 101.84 | 90.35 |
|  | <40% | ≥70% | 0.78 | 0.22 | 0.60 | 0.23 | 19.42 | <0.0001* | 91.55 | 58.15 |
| Satellite taxa | <40% | 40-69% | 0.34 | 0.21 | 0.34 | 0.22 | 0.002 | 0.968 | 120.65 | 120.29 |
|  | 40-69% | ≥70% | 0.34 | 0.22 | 0.32 | 0.20 | 0.607 | 0.436 | 100.52 | 93.57 |
|  | <40% | ≥70% | 0.34 | 0.21 | 0.32 | 0.20 | 0.632 | 0.427 | 81.67 | 75.65 |
