# Supplementary material for: Lung function and microbiota diversity in cystic fibrosis
Source: Microbiome. 2020 Apr 2;8:45. doi: 10.1186/s40168-020-00810-3 (PMC7114784; doi:10.1186/s40168-020-00810-3)
Supplement: Supplementary file 5 — Additional file 4: Figure S1. Measures of Hedges’ d effect size based on comparisons of (A) diversity and (B) dominance in the microbiota, core taxa, and satellite taxa, when stratified into lung disease categories. Columns represent the effect size and error bars represent the standard error of effect size. Standard error bars that cross zero indicate no significant effect on diversity or dominance between lung disease categories. In each instance, within (A) positive effect sizes represent higher diversity in the second of the two lung disease categories being compared. Within (B) negative effect sizes represent lower dominance in the 2nd of the two lung disease categories being compared. Measures of diversity and dominance when stratified by lung disease category are presented in Fig. 3a and b, respectively. [file 40168_2020_810_MOESM4_ESM.docx]

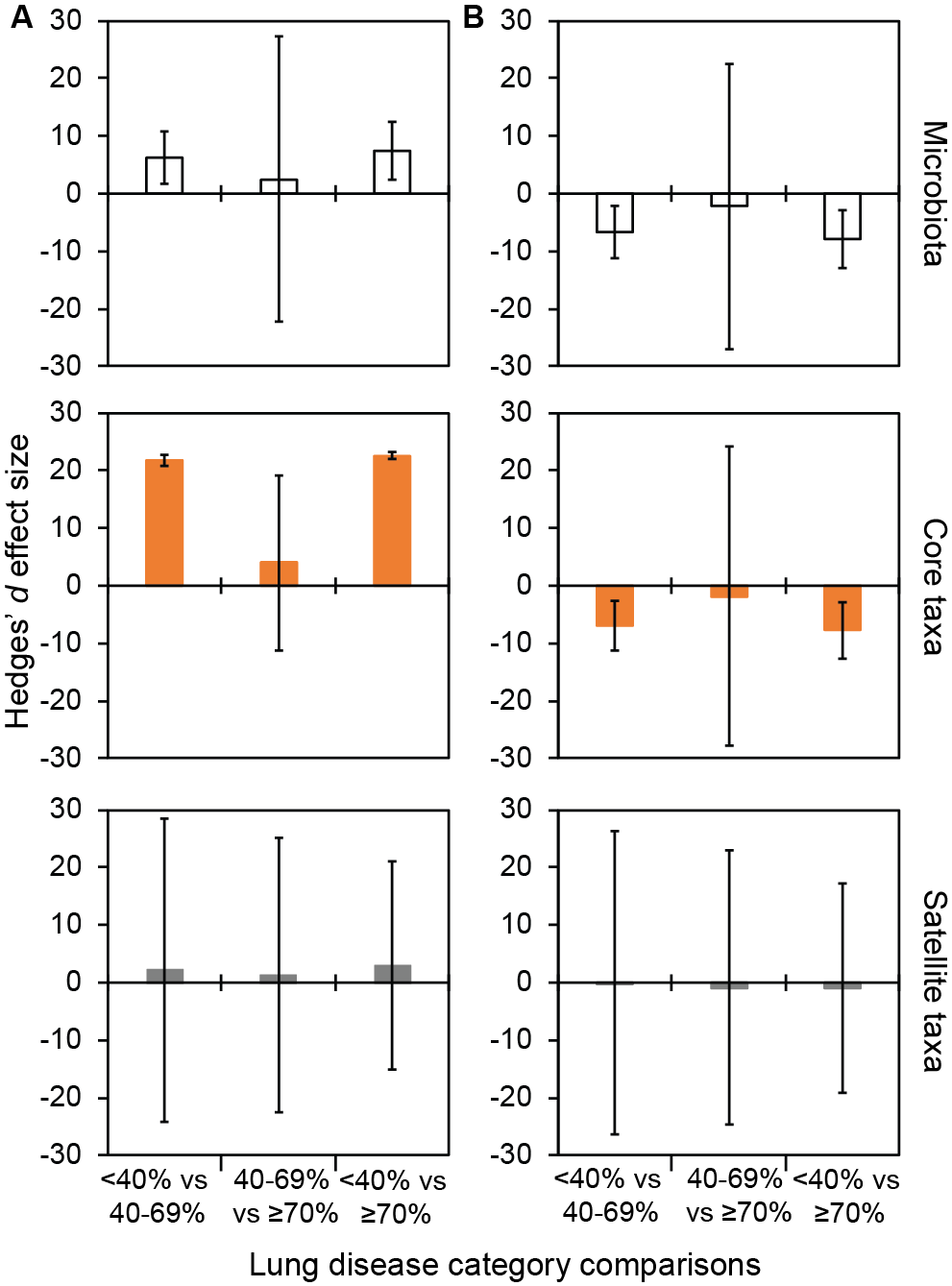


**Figure S1** Measures of Hedges’ *d* effect size based on comparisons of (A) diversity and (B) dominance in the microbiota, core taxa, and satellite taxa, when stratified into lung disease categories. Columns represent the effect size and error bars represent the standard error of effect size. Standard error bars that cross zero indicate no significant effect on diversity or dominance between lung disease categories. In each instance, within (A) positive effect sizes represent higher diversity in the second of the two lung disease categories being compared. Within (B) negative effect sizes represent lower dominance in the 2^nd^ of the two lung disease categories being compared. Measures of diversity and dominance when stratified by lung disease category are presented in Figure 3a and b, respectively.
