# Supplementary material for: Lung function and microbiota diversity in cystic fibrosis
Source: Microbiome. 2020 Apr 2;8:45. doi: 10.1186/s40168-020-00810-3 (PMC7114784; doi:10.1186/s40168-020-00810-3)
Supplement: Supplementary file 6 — Additional file 5: Table S4. PERMANOVA summary statistics from testing for significant differences in microbiota composition between lung function categories. Given in each instance are mean Bray-Curtis similarity within and between categories (± standard deviation of the mean), F-statistic, and significance (P). Asterisks denote significant differences in composition following one-way PERMANOVA tests with Bonferroni correction. [file 40168_2020_810_MOESM5_ESM.docx]

**Table S4** PERMANOVA summary statistics from testing for significant differences in microbiota composition between lung function categories. Given in each instance are mean Bray-Curtis similarity within and between categories (± standard deviation of the mean), *F*-statistic, and significance (*P*). Asterisks denote significant differences in composition following one-way PERMANOVA tests with Bonferroni correction.

|  |  |  | Category 1 |  | Category 2 |  | Between Categories | | |  |
| --- | --- | --- | --- | --- | --- | --- | --- | --- | --- | --- |
|  | Category 1 | Category 2 | Mean | ±SD | Mean | ±SD | Mean | ±SD | *F* | *P* |
| Microbiota | <40% | 40-69% | 0.37 | 0.11 | 0.39 | 0.12 | 0.37 | 0.10 | 5.26 | 0.0006* |
|  | 40-69% | ≥70% | 0.39 | 0.12 | 0.40 | 0.13 | 0.39 | 0.10 | 10.48 | 0.0003* |
|  | <40% | ≥70% | 0.37 | 0.11 | 0.40 | 0.13 | 0.37 | 0.10 | 3.01 | 0.0165* |
| Core taxa | <40% | 40-69% | 0.75 | 0.16 | 0.33 | 0.28 | 0.34 | 0.31 | 8.52 | 0.0003* |
|  | 40-69% | ≥70% | 0.33 | 0.28 | 0.29 | 0.25 | 0.30 | 0.25 | 13.54 | 0.0003* |
|  | <40% | ≥70% | 0.75 | 0.16 | 0.29 | 0.25 | 0.27 | 0.28 | 3.38 | 0.0201* |
| Satellite taxa | <40% | 40-69% | 0.24 | 0.13 | 0.29 | 0.13 | 0.25 | 0.11 | 4.73 | 0.0003* |
|  | 40-69% | ≥70% | 0.29 | 0.13 | 0.31 | 0.15 | 0.29 | 0.12 | 4.37 | 0.0003* |
|  | <40% | ≥70% | 0.24 | 0.13 | 0.31 | 0.15 | 0.25 | 0.11 | 1.99 | 0.0003* |
